# T-Cell Engaging Antibodies in Diffuse Large B Cell Lymphoma—An Update

**DOI:** 10.3390/jcm12216737

**Published:** 2023-10-25

**Authors:** Shalini Balendran, Constantine Tam, Matthew Ku

**Affiliations:** 1 Alfred Health, Melbourne, VIC 3004, Australiaconstantine.tam@alfred.org.au (C.T.); 2 St. Vincent’s Hospital, Melbourne, Fitzroy, VIC 3065, Australia

**Keywords:** bispecific antibodies, DLBCL, refractory, relapsed, CART, cytokine release syndrome, neurotoxicity

## Abstract

Novel cellular immunotherapies such as T-cell engaging antibodies (TCEAbs) are changing the landscape of treatment for diffuse large B cell lymphoma (DLBCL), especially in the relapsed/refractory (R/R) setting. TCEAbs harness the power of the host immune system to induce killing of tumor cells by binding to both the tumor antigen and the T-cell receptor. Since the approval of blinatumomab for R/R acute lymphoblastic leukemia, there has been significant development in novel TCEAbs. Many of these novel TCEAbs have shown promising effectiveness in R/R DLBCL, with favorable response rates including complete remissions, even in heavily pretreated patients. There are unique therapy-related toxicities with TCEAbs, namely cytokine release syndrome (CRS) and immune effector cell-associated neurotoxicity (ICANS), and it is important to both recognize and manage these side effects appropriately. This review examines the development and mechanism of action of these TCEAbs, and the available published data from clinical trials. Their role in the treatment of DLBCL, the management of therapy-related adverse events, and the mechanisms of resistance will also be discussed.

## 1. Introduction

Diffuse large B-cell lymphoma (DLBCL) is the most common non-Hodgkin lymphoma (NHL) subtype [1,2]. Frontline chemoimmunotherapy results in a cure rate of approximately 60%, and for patients with relapsed/refractory DLBCL the standard treatment has been salvage chemotherapy, followed by autologous stem cell transplantation (ASCT) in transplant eligible patients. Due to patient factors, treatment intensity or chemo-refractoriness, only a small minority of patients are cured by this approach and the majority of patients with R/R DLBCL have a poor prognosis, especially those with primary refractory disease or those that are not ASCT candidates [3,4,5]. This represents an area of unmet medical need.

The introduction of T cell directed immune effector cell therapies has revolutionized the treatment paradigms for R/R DLBCL, demonstrating durable efficacy with a tolerable safety profile in this heavily pretreated patient cohort [6,7,8,9,10,11,12]. Both autologous chimeric antigen receptor (CAR) T therapy and T-cell engaging antibodies (TCEAbs) harness the patients’ own T cells to eliminate the malignant cells. Recent published data has shown the significant superiority of both axicabtagene ciloleucel and lisocabtagene maraleucel compared to standard salvage treatments. While CAR T has now been embedded as an accepted salvage option in R/R DLBCL, there are shortcomings such as accessibility, treatment related toxicities, and the time needed for manufacturing. High risk features such as a rapid tempo of disease progression would often preclude patients from receiving autologous CAR T. TCEAbs are “off the shelf” antibody-based treatments that do not require manufacturing, bridging therapy, or lymphodepleting chemotherapy. The promising effectiveness so far with the TCEAbs in R/R DLBCL, in addition to the apparent lower rates of toxicities compared to CAR T, has seen the TCEAbs being studied in the frontline DLBCL setting, often combined with chemoimmunotherapy. Patients being treated with either CAR T or TCEAbs can develop cytokine release syndrome (CRS) and/or immune effector cell-associated neurotoxicity syndrome (ICANS). As more patients are treated with these immunotherapies, it has become necessary to recognise and manage these unique toxicities optimally.

This review will focus on the main TCEAbs currently under development in R/R DLBCL, including their structures, mechanisms of action, efficacy, and safety data. This review will also discuss the role TCEAbs will have in the overall DLBCL management landscape and how to best manage toxicities associated with them.

## 2. Basic Scientific Mechanism

**Figure 1:**
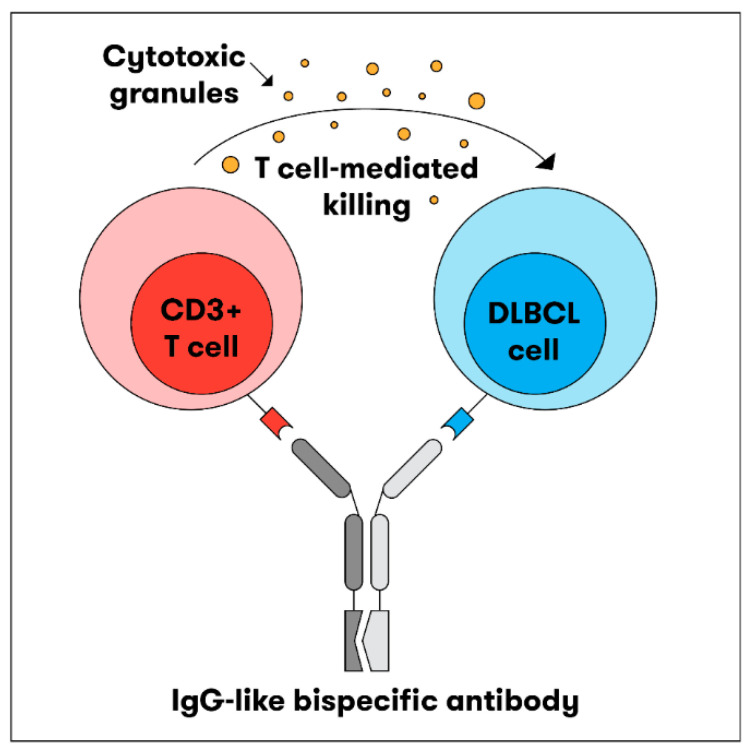
Bispecific antibody mechanism of action. The BsAb simultaneously binds the tumour antigen and CD3 epsilon on the surface of a T-cell, resulting in tumour cell killing.

TCEAbs harness the power of the immune system to induce killing of tumour cells. In general, TCEAbs bind to both the tumour antigen and T-cell receptor to form an immunologic synapse and promote cell-mediated cytotoxicity in a major histocompatibility complex (MHC)-independent fashion. This is particularly important in DLBCL, as fifty percent of cases lack MHC class I cell-surface expression secondary to genetic aberrations, resulting in immune evasion. The specific mechanism of action can vary depending on the design of the antibody, which is discussed in further detail below [13,14,15] (Figure 1).

## 3. Structure: TCEAbs Can Be Divided into Bispecific (BsAbs) and Trispecific Products (TsAbs)

### 3.1. Bispecific Antibodies

As the name implies, BsAbs are designed to bind two different targets simultaneously. A BsAb may bind to either CD19 or CD20, antigens expressed on the surface of B-cells, and CD3, an antigen expressed on T-cells. By binding to CD19 or CD20 and CD3, the BsAb can bring the T-cells in close proximity to the DLBCL cells, resulting in the activation of T-cells, release of perforin/granzymes, and lysis of the target CD20-expressing cell. BsAbs can be constructed using two different formats, fragment based BsAb (“non-IgG-like”) and fragment crystallizable (Fc) BsAb (“IgG-like”) [16].

#### 3.1.1. Non-IgG-like BsAbs

This subclass includes bispecific T-cell engagers, dual-affinity re-targeting antibodies (DARTs), and tetravalent tandem diabodies (TandAb) [17].

Currently, the most used BsAb in this subclass are bispecific T-cell engagers (e.g., blinatumomab). The structure consists of two single-chain variable fragments (scFvs) that are fused with one polypetide linker. Each scFV has heavy (VH) and light (VL) chains. DARTs similarly contain two VL-VH pairs but have two distinct polypetide chains, which are stabilized by an interchain disulphide bridge. TandAbs are manufactured as a single polypeptide chain, requiring chain dimerization to link four antibody VH and VL chains. This produces a tetravalent bispecific molecule (four antigen-recognition sites) for two different antigens [18,19]. These antibodies lack the constant Fc region and consequently have a short half-life due to the high rate of renal clearance. However, they have better tumour penetration secondary to their small size and are easier to manufacture when compared to IgG-like antibodies [20]. Additionally, they do not exhibit antibody-dependent cell-mediated cytotoxicity (ADCC), antibody-dependent cellular phagocytosis (ADCP), or complement fixation due to absence of the Fc-portion [17].

#### 3.1.2. IgG-like BsAbs

The majority of BsAbs under investigation in DLBCL clinical trials have full-length, IgG-like configurations with Fc domains, resembling native immunoglobulins. There are various technologies used to manufacture them, such as knobs-into holes heterodimerization, head-to-tail fusion, and controlled fragment antigen-binding (Fab)-arm exchange [16]. The presence of an Fc region prevents IgG catabolism and is responsible for the longer half-life of these BsAbs. Thus, continuous infusion is not required. While long lasting anti-tumour effect is clearly advantageous, the potential downside is Fc-mediated toxicity, specifically CRS via antibody-dependent FcγR-mediated crossbinding of CD3 and T cells. In order to counteract this effect, the Fc domain is mutated during the engineering process to reduce toxicity but maintain drug half-life in vivo [21].

### 3.2. Trispecific Antibodies (TsAbs)

Novel T-cell redirecting TsAbs are postulated to be superior to BsAbs due to the presence of three different antigen binding sites on cytotoxic T lymphocytes (CTLs) and malignant B-cells. Only two clinical drug trials currently exist in DLBCL, and both are first-in-human, phase I studies with no results available yet. Both TsAbs under investigation are IgG-like in structure [22,23].

## 4. Clinical Efficacy

### 4.1. CD19-Directed BsAb

#### Blinatumomab

Blinatumomab is an anti-CD3 x anti-CD19 bispecific T-cell engager and was the first BsAb approved for B-cell malignancies. It is administered as a continuous intravenous infusion due to its reduced half-life (2 h) and was initially approved in R/R Ph-B-ALL. The evidence supporting this indication was primarily derived from the TOWER study, which compared blinatumomab monotherapy with ‘standard-of-care chemotherapy’ [24]. Pivotal results seen in the 2018 BLAST trial resulted in expansion of FDA approval to include treatment of minimal residual disease positive B-ALL in patients in complete haematological remission [25].

Following unprecedented success in B-ALL, blinatumomab was subsequently trialed as salvage therapy in R/R NHL [26]. A phase 2 study by Coyle et al. evaluated single agent blinatumomab as second salvage therapy in R/R NHL. The trial enrolled 41 patients, including 34 patients with DLBCL, treated with stepwise blinatumomab (9–28–112 µg/day). Following 12 weeks of treatment, the ORR was 37% with a CR of 22%. However, there was a high rate of treatment discontinuation, with only 59% receiving 80% of the intended dose, largely due to disease progression. Grade 3 neurological events occurred in 24%, all of which resolved [27]. Another phase 2 study conducted by Viardot et al. investigated stepwise blinatumomab (9–28–112 µg/day; *n* = 23) or flat dosing (112 μg/day; *n* = 2) exclusively in R/R DLBCL. Similar findings were demonstrated with an ORR in 43% and CR in 19% of the 21 evaluable patients. Grade 3 neurotoxicity occurred in 22% in the stepwise cohort and in both patients in the flat dose cohort [28].

More recent trials have studied blinatumomab in combination with lenalidomide (NCT02568553), and as consolidation treatment post-autologous stem cell transplant (ASCT) in patients with DLBCL (NCT03072771). In patients receiving blinatumomab and lenalidomide (*n* = 12), the ORR was 83% (50% CR) at a median follow-up time of 14.3 months [29]. Blinatumomab consolidation therapy post-ASCT (*n* = 10), showed 60% of patients remained in remission at a median follow-up of 14.5 months [30].

The emergence of IgG-like BsAbs with easier administration and less neurotoxicity have overshadowed further development of blinatumomab in the setting of DLBCL.

### 4.2. CD20-Directed BsAb

At the time of writing, ten CD20/CD3 BsAbs are under clinical trial investigation in DLBCL. Agents that are in phase 1/2 include odronextamab (REGN1979), plamotamab (XmAb13676), imvotamab (IgM-2323), GB261, TQB2825, CM355, and EX103. Mosunetuzumab (RG7828), epcoritamab (GEN3013), and glofitamab (RO7082859) are entering phase 3 for treatment of R/R DLBCL

#### 4.2.1. Mosunetuzumab

Mosunetuzumab is a fully humanised IgG1, CD20 × CD3 BsAb, which was developed using knobs-into-holes technology. It has been produced in both IV and SC formulations with an estimated half-life of 6–11 days. It was the first IgG-like BsAb engineered and has been evaluated in both indolent (iNHL) and aggressive B-cell NHL (aNHL) as a monotherapy or combined with other agents.

Initial results of single-agent mosunetuzumab were obtained in the phase 1 dose escalation GO29871 trial in R/R B-NHL. The BsAb was administered for up to 8 cycles in patients who achieved CR and up to 17 cycles in those with partial response or stable disease. In total, 197 patients were included; 68 patients with iNHL and 129 patients with aNHL. High response rates were seen in iNHL with an ORR of 62.7% (CR 43.3%), whereas results were less impressive in aNHL with an ORR of 37.4% (CR 19.5%). AEs were predominantly reported in the first cycle and the majority were related to neutropenia (28.4%), CRS (27.4%), or hypophosphataemia (23.4%). All CRS events resolved and were mostly low-grade (1% grade 3) [31].

Clinical efficacy of mosunetuzumab has been more noteworthy in indolent lymphoma. As a result, it is unlikely that it will be developed as a single agent for aggressive lymphomas.

#### 4.2.2. Epcoritamab

Epcoritamab is a subcutaneously (SC) administered IgG1, CD20 × CD3 BsAb. It is manufactured by controlled Fab-arm exchange technology and has a half-life of 8.7 days.

The EPCORE NHL-1 study phase 1/2 study tested single-agent epcoritamab in subjects with R/R B-NHL. SC epcoritamab was administered in 28-day cycles until disease progression or unacceptable toxicity. The recommended phase 2 dose was 48 mg, and the maximum tolerated dose was not reached [32]. The dose-expansion cohort evaluated 157 patients who were heavily pre-treated; 71% had at least three prior lines of therapy and 39% had previous CAR T therapy. The dataset demonstrated high ORR (63%) and CR rate (39%). Amongst the 157 patients enrolled in the study, 51 remained on treatment. The main reason for discontinuation was secondary to progressive disease rather than adverse events (AEs). Most treatment-emergent AEs were low grade and occurred early in treatment (cycle 1–3). The most common AE was CRS in 49.7% of patients, of which only 2.5% were grade 3. Immune effector cell-associated neurotoxicity syndrome (ICANS) was not a frequent event and occurred in ten patients (6.4%); nine were grade 1–2 and resolved [33]. Durable complete remissions in DLBCL have been reaffirmed in longer follow-up from the EPCORE NHL-1 trial. Median duration of CR was 20.8 months and median time to CR was 2.7 months [34].

Phase 3 studies investigating epcoritamab in R/R and newly diagnosed DLBCL are currently underway. EPCORE DLBCL-1 is an ongoing multicentre, randomized trial evaluating the efficacy of SC epcoritamab versus either rituximab, gemcitabine, and oxaliplatin (R-GemOx) or bendamustine and rituximab (BR). Patients must have failed or be ineligible for ASCT in the setting of R/R DLBCL. In subjects with newly diagnosed, high-risk (IPI 3–5) DLBCL, the EPCORE DLBCL-2 study evaluates the safety and preliminary efficacy of epcoritamab in combination with R-CHOP compared to R-CHOP alone. Approximately 900 patients will be randomized 2:1 to either combination treatment or R-CHOP alone, including double-hit and triple-hit patients.

#### 4.2.3. Glofitamab

Glofitamab is an intravenous IgG1 BsAb that binds bivalently to CD20 on B cells and monovalently to CD3 on T cells. It has a novel 2:1 format that is engineered by head-to-tail fusion technology and has a half-life of 10 days [35].

A phase 1/2 trial conducted by Hutchings et al. studied glofitamab monotherapy in 171 patients with R/R B-cell NHL. Before commencing glofitamab, patients received a single dose of obinutuzumab pretreatment (1 × 1000 mg) to mitigate CRS. Fixed dose (0.6–25 mg) or step-up glofitamab dosing (target dose: 16 mg or 30 mg) was subsequently administered every 2–3 weeks. Encouraging response rates were observed, with an ORR of 48% and CR of 33.1% in aggressive NHL patients.

In the phase 2 part of the clinical trial (dose-expansion), Dickinson et al. enrolled 154 patients with R/R DLBCL who had received at least two lines of previous therapy. A fixed treatment duration, 12 cycles of glofitamab monotherapy, was administered every three weeks. Step-up glofitamab dosing involved 2.5 mg on day 8 cycle 1, 10 mg on day 15 cycle 1, and 30 mg on day 1 of cycle 2 to cycle 12. ORR and CR rates were 59% and 38%, respectively, similar to phase 2 epcoritamab data. CR was achieved early, reported as a median time of 43 days. In patients with a complete remission, 66% were in ongoing CR after a median follow-up of 18.3 months. Additionally, a high proportion of patients remained progression free at 12 months post-end of treatment (PFS rate: 80%, OS rate: 94%). Glofitamab was well tolerated with a low rate of discontinuation secondary to AEs, occurring in only 9% of patients. CRS was the most frequent AE (64%); however, it was generally low-grade. The most common grade 3/4 AE was neutropenia [36].

Several other trials are ongoing, assessing glofitamab monotherapy and combination therapy in DLBCL. A phase 1b/2 trial is investigating glofitamab and polatuzumab vedotin in R/R NHL. Polatuzumab is an antibody drug conjugate, composed of anti-CD79b monoclonal antibody conjugated to monomethyl auristatin E (microtubule inhibitor). CD79b is located on normal B-cells and most mature B-cell malignancies, hence it is a good target for patients’ refractory to standard chemotherapy. All study patients received a single dose of obinutuzumab on day 1, followed by polatuzumab (1.8 mg/kg) on day 2, and step-up glofitamab from day 8. Glofitamab was combined with polatuzumab for 6 cycles and administered as a single agent from cycle 7–12. A 13 month follow up (data cutoff 25 January 2023) reported high response rates (ORR of 78% and CR of 56%). Duration of complete response at 12 months was 73.1% [37]. Combinations with platinum-based chemoimmunotherapy are also being explored, including a randomized phase 3 study of gemcitabine-oxaliplatin +/− glofitamab.

#### 4.2.4. Odronextamab

Odronextamab is a fully human IgG4-based, CD20 × CD3, hinge-stabilised BsAb. It is administered by either SC or IV and has a half-life of 14 days in cynomolgus monkeys [38].

In the ELM-1 trial, a multicentre phase 1 study, IV odronextamab monotherapy was administered to 145 patients with R/R B-NHL. As with other BsAbs, odronextamab was dosed in a step-up schedule with steroid premedication in cycle 1, followed by once per week in cycles 2–4, and then every two weeks until disease progression. Patients were heavily pre-treated (median of three previous therapies) and 29% of patients had received previous CAR T therapy. In patients with FL who received ≥ 5 mg, the ORR was 91% and CR was 72%. Among patients with DLBCL without prior CAR T-cell therapy who received ≥ 80 mg, the ORR was 53% (all responses were CR) and 33% (CR 27%) in those who had previous CAR T-cell therapy. While treatment-emergent AEs were common (93%), odronextamab had a manageable safety profile. Grade ≥3 CRS occurred in 6% of patients and grade ≥3 neurological events in 3%. The dose-expansion portion of this study is ongoing [39].

The phase 2 trial (ELM-2) of odronextamab monotherapy in R/R DLBCL studied 140 DLBCL patients with a minimum of two prior treatment lines. As of 15 September 2022, ORR and CR rates were 49% and 31%, respectively. Similar responses were seen in patients who had prior CART therapy. Interestingly, the duration of CR at 18 months was 48%, which appears to be less promising than results observed in glofitamab and epcoritamab. Further follow-up will be required [40].

#### 4.2.5. Plamotamab

Plamotamab is an intravenous IgG1 bispecific anti-CD20/CD3 antibody, produced through an XmAb protein engineering platform [19].

The phase 1 dose-escalation study investigating plamotamab monotherapy in patients with R/R NHL is ongoing. The recommended dose safety/efficacy update and escalation exposure-response analysis by Patel et al. included 36 patients enrolled before April 2022. The median number of prior lines of therapy was four and 50% of patients had previously received CAR-T. At the recommended dose, 19 patients with DLBCL had an ORR of 47.4% and CR rate of 26.3%. The most common AE was CRS, occurring in 72.2% of patients, and usually occurred in C1. No grade ≥3 CRS was reported [41].

A phase 2 multicentre study is in progress evaluating plamotamab combined with tafasitamab and lenalidomide vs. tafasitamab and lenalidomide doublet in R/R DLBCL [42].

### 4.3. Trispecific Antibodies

#### 4.3.1. JNJ-80948543

JNJ-80948543 is a trispecific monoclonal antibody that promises to be the next breakthrough in the treatment of B-NHL due to two separate B-cell binding sites. The SC TsAb binds the CD3 antigen on T-cells, and both CD79b and CD20 on B-cells, resulting in cytolysis of malignant B-cells.

A global phase 1 study of the TsAb in patients with NHL and CLL is ongoing to determine the recommended phase 2 dose and optimal dosing schedule [22].

#### 4.3.2. PIT565

PIT565 is an anti-CD19, anti-CD3, and anti-CD2 TsAb. Targeting CD2 on T cells is postulated to overcome T-cell exhaustion, which may contribute to treatment failures seen in CAR-T therapies or BsAbs. A first-in-human multicentre trial of PIT565 is in progress in patients with R/R B-NHL and R/R B-ALL [23].

## 5. Role in DLBCL Management

As a class, TCEAbs have been studied both as initial DLBCL treatment, as well as in the R/R setting. They have been used as monotherapy and in combination with other therapeutics, such as chemotherapy or immunomodulatory agents. Based on positive trial results, single agent glofitamab and epcoritamab have recently been granted FDA approval for the treatment of R/R DLBCL and high-grade B-cell lymphoma (≥2 prior lines of systemic therapy). Glofitamab has also received approval from the European Medicines Agency (EMA) for the same patient cohort. Consequently, the current role of these TCEAbs appears to be in the R/R setting. Nonetheless, due to the likelihood of TCEAbs having increased efficacy when used earlier in the treatment algorithm, there are ongoing trials examining their utility in the upfront setting. If positive signals are observed, TCEAbs will potentially be integrated in the first line setting in the future.

In the combination studies, so far, the data suggest that the addition of TCEAbs to the chemotherapy backbone has not resulted in significant treatment interruption or new toxicities. Despite encouraging data with these TCEAbs, long-term efficacy and safety results are required to inform their role in DLBCL management, especially in terms of treatment sequencing with the other therapeutic agents. Patient selection, optimal timing of treatment, and combination regimens are areas of uncertainty that will need to be addressed. Given the efficacy of RCHOP in newly diagnosed DLBCL, and more recent data showing improved PFS with polatuzumab-RCHP [43], TCEAbs are likely to have more utility in high risk patients in the upfront setting (e.g., high IPI scores), or as bridging therapy in chemorefractory patients prior to CAR T. In the R/R setting, it will be vital to clarify sequencing of ASCT, CAR T, and TCEAbs, and their potential combination. With recent data suggesting the superiority of CAR T versus ASCT, it has been preferenced in many countries [9,44]. The available data of CAR T failure “rescue” by TCEAbs is fascinating; however, the inverse is uncertain. Furthermore, until the data on durability of response are known (apart from glofitamab), CAR T would appear to be ahead in terms of curative potential. It would also be imperative to elucidate the optimal duration of TCEAbs to maximize the DOR, but also to minimize treatment related toxicities. Future study designs should ideally include fixed duration of treatment as well as retreatment strategy on relapse.

## 6. Management of TCEAb Mediated Toxicities

The search for biomarkers that predict TCEAb efficacy, toxicity, and resistance is ongoing, and will likely guide future decision making. The most unique side effects of TCEAbs, such as CAR T therapy, are CRS and ICANS. CRS is an immunotherapy-induced systemic inflammatory syndrome that occurs due to the hyperactivation of the immune system that results in the release of multiple cytokines, including TNFα, IL6, IL10, and IFN. Although CRS is often the most prominent TCEAb related toxicity, it appears to occur less frequently and to be of lesser severity when compared to CAR T. Fever, hypotension, and hypoxia are the hallmarks of CRS, as per the American Society for Transplantation and Cellular Therapy (ASTCT) guidelines, but other symptoms may occur such as myalgias, skin rash, arthralgias, and headache. It is important to recognize CRS early on and manage accordingly as progression to more severe CRS can occur rapidly, with deterioration resulting in multiorgan failure and the need for circulatory and respiratory support in the intensive care setting [45,46]. Due to the immunocompromised nature of the patients receiving TCEAbs, concurrent infections can complicate the CRS management, and patients should be treated for both empirically without delay. Other conditions that could mimic CRS include infusion related reaction, haemophagocytic lymphohistiocytosis, and macrophage activation syndrome [47]. Features of high disease burden have been found to be correlative to the occurrence of higher-grade CRS, including advanced stage disease, circulating lymphoma cells, bulky disease, and bone marrow involvement [48,49,50]. Many different mitigation approaches have been designed apart from premedications with acetaminophen, antihistamine, and steroid, such as tumor bulk reduction prior to TCEAb, step up dosing in the first cycle, and the use of dexamethasone. Due to the structure of glofitamab with its two antiCD20 domains, it is possible to administer obinutuzumab seven days prior to the first dose in order to reduce CRS [51]. As an antiCD20 monoclonal antibody, obinutuzumab can reduce peripheral circulating B cells and lower T cell hyperactivation, cytokine release, and endothelial cell activation. The management of CRS depends on the ASTCT grading. Milder grade CRS is usually initially managed by supportive treatment with TCEAb interruption, IV hydration, steroid, and acetaminophen. More severe grade CRS despite initial therapy will require treatment with tocilizumab, an anti-IL6 antibody, as well as intensive care unit intervention for inotropic and respiratory support.

ICANS can occur as manifestation of central nervous system (CNS) deficits. It is frequently coupled with CRS, and again is the result of T cell hyperactivation. Patients can present with a range of symptoms from headache, delirium, dysgraphia, dysphasia, tremor, and concentration difficulty to seizures or even encephalopathy [46,52,53]. Although relatively common with blinatumomab (G3 or higher in 24% in a phase 2 trial of aggressive NHL), it is much less frequent with the newer TCEAbs. As a therapeutic class, TCEAbs have a much lower incidence of significant ICANS when compared to CAR T. The ASTCT guidelines for ICANS use an Immune Effector Cell-Associated Encephalopathy (ICE) screening score to assess the extent of encephalopathy, which is added to the other neurological features of ICANS (e.g., seizure, motor findings) in the final grading. It is vital to recognize ICANS early as sometimes the signs can be subtle, and to provide appropriate management to avoid complications. Specific investigations for ICANS would include neuroimaging, EEG, fundoscopy, and lumbar puncture, and it might be necessary to repeat these. Supportive strategies are pivotal, including antiepileptic medications for seizures, neurological monitoring, aspiration preventions, and intensive care unit support for ventilation if needed. The mainstay of treatment is steroid e.g., dexamethasone or methylprednisolone, which is given at high doses and tapered when there is clinical improvement. Tocilizumab is only used if CRS occurs concurrently with ICANS; otherwise, it is not used for ICANS alone as its access to the CNS is limited.

The data on the management of refractory/resistant immunotherapy related toxicities are scant, and consist of preclinical data, case series, or institutional reports. The pharmacological interventions being studied include siltuximab (an IL6 antagonist), anakinra (an IL1 receptor antagonist), cyclophosphamide, ruxolitinib (JAK inhibitor), and IVIG. Currently there are no standardized strategies for the management of tocilizumab and/or steroid resistant CRS/ICANS, and more research is required in this space to provide evidence-based guidelines. Furthermore, with the administration of further immunosuppressants, increased risk of sepsis should be considered, and appropriate surveillance and prophylaxis utilized.

## 7. Mechanisms of Resistance

Therapy failure due to resistance remains an important issue. Several potential mechanisms of resistance have been proposed, such as tumor surface antigen loss, acquisition of new oncogenic mutations, or T cell exhaustion or dysfunction via persistent TCR activation [32,51,54,55,56,57,58,59,60]. Different approaches have been designed to attempt to prevent or overcome these mechanisms, such as targeting multiple tumor antigens simultaneously to avoid antigen loss or combination with other immunomodulatory agents to enhance T cell activity. Another strategy is to utilize the TCEAbs early in the treatment course, in an attempt to avoid T cell exhaustion or new oncogenic mutations. More research is needed to study and overcome these potential pathways of resistance.

## 8. Conclusions

Novel immunotherapies such as TCEAbs have revolutionized the treatment landscape in DLBCL. There have been rapid and substantial breakthroughs in their development since the advent of blinatumomab. Currently, there is an impressive number of TCEAbs demonstrating promising efficacy and safety data, leading to the recent approval of certain TCEAbs by the registration authorities in R/R DLBCL. The lower rates of CRS and ICANS of TCEAbs, together with the lack of manufacture time and potential effectiveness in rescuing CAR T failures, mean that they should have an important role in the future alongside CAR T therapy. Furthermore, if significant efficacy is observed in the first line studies then TCEAbs can potentially improve the cure rate of DLBCL as part of upfront treatment.

This review examined the various TCEAbs in DLBCL, from their mechanisms of action to the published clinical trial data. There are still many unanswered questions, including the optimal treatment sequencing of these TCEAbs, the best mode of administration, monotherapy vs. combination therapy, and prevention of resistance. This will undoubtedly become clearer with more experience and future clinical trials (Table 1).

## Figures and Tables

**Table 1:** Overview of BsAbs.

Drug Name	Structure	Epitopes (Ratio)	Format	½ Life	Technology	Administration/Schedule
Blinatumomab [27]	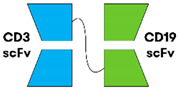	CD19 × CD3	IgG1	2.1 h	BiTE^®^ (Amgen, Southend Oaks, CA, USA)	IV 4W continuous infusion (2W treatment-free interval)
Mosunetuzumab [16,31]	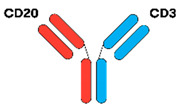	CD20 × CD3	IgG1	6–11 days	Knobs-into-holes	IV or SC 8–17 cycles SUD C1–C2 3W C3 onwards
Epcoritamab [16,33]	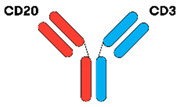	CD20 × CD3	IgG1	8.7 days	Controlled Fab-arm exchange	SC SUD C1 1W C1–C3. 2W C4–9. 4W C10 onwards
Glofitamab [16,36]	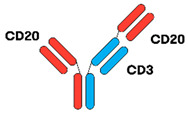	(CD20)_2_ × CD3	IgG1	10 days	Head-to-tail fusion	IV 12 cycles 1 × obinutuzumab (1000 mg) 7 days prior to first dose of glofitamab
Odronextamab [16,39]	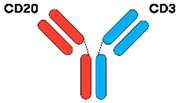	CD20 × CD3	IgG4	14 days	VelociSuite^®^ (Regeneron, Tarrytown, NY, USA)	IV or SC SUD C1 W1 C2–C4 W2 C5 onwards
Plamotamab [16,41]	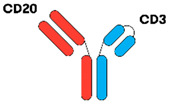	CD20 × CD3	IgG1	NR	XmAb^®^ (Amgen, Southend Oaks, CA, USA)	IV or SC Weekly dosing
Imvotamab [16,61]	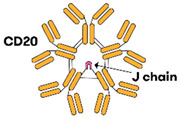	(CD20)_10_ × CD3	IgM	NR	IGM biosciences^®^ (IGM biosciences, Mountain View, CA, USA)	IV Weekly or 3W dosing

C, cycles; IgG, immunoglobulin G; IgM, immunoglobulin M; IV, intravenous; NR, not reported; SC, subcutaneous; SUD, step-up-dosing; W, weekly.

## Data Availability

Not applicable.

## References

[1] Morton L.M., Wang S.S., Devesa S.S., Hartge P., Weisenburger D.D., Linet M.S. (2006). Lymphoma incidence patterns by WHO subtype in the United States, 1992–2001. Blood.

[2] Swerdlow S.H., Campo E., Pileri S.A., Harris N.L., Stein H., Siebert R., Advani R., Ghielmini M., Salles G.A., Zelenetz A.D. (2016). The 2016 revision of the World Health Organization classification of lymphoid neoplasms. Blood.

[3] Crump M., Neelapu S.S., Farooq U., Van Den Neste E., Kuruvilla J., Westin J., Link B.K., Hay A., Cerhan J.R., Zhu L. (2017). Outcomes in refractory diffuse large B-cell lymphoma: Results from the international SCHOLAR-1 study. Blood.

[4] Salles G.A., Pettengell R., Cordoba R., Dlugosz-Danecka M., Jurczak W., Tilly H. (2019). Treatment of aggressive B-cell non-Hodgkin lymphoma beyond frontline therapy in patients not eligible for stem cell transplantation: A structured review. Leuk Lymphoma.

[5] Jabbour E., Chalhoub B., Suzan F., Aloulou S., Cainap C., Toumi N., Ferme C., Carde P., Ribrag V. (2004). Outcome of elderly patients with aggressive Non-Hodgkin’s lymphoma refractory to or relapsing after first-line CHOP or CHOP-like chemotherapy: A low probability of cure. Leuk Lymphoma.

[6] Neelapu S.S., Locke F.L., Bartlett N.L., Lekakis L.J., Miklos D.B., Jacobson C.A., Braunschweig I., Oluwole O.O., Siddiqi T., Lin Y. (2017). Axicabtagene Ciloleucel CAR T-Cell Therapy in Refractory Large B-Cell Lymphoma. N. Engl. J. Med..

[7] Schuster S.J., Svoboda J., Chong E.A., Nasta S.D., Mato A.R., Anak O., Brogdon J.L., Pruteanu-Malinici I., Bhoj V., Landsburg D. (2017). Chimeric Antigen Receptor T Cells in Refractory B-Cell Lymphomas. N. Engl. J. Med..

[8] Abramson J.S., Palomba M.L., Gordon L.I., Lunning M.A., Wang M., Arnason J., Mehta A., Purev E., Maloney D.G., Andreadis C. (2020). Lisocabtagene maraleucel for patients with relapsed or refractory large B-cell lymphomas (TRANSCEND NHL 001): A multicentre seamless design study. Lancet.

[9] Locke F.L., Miklos D.B., Jacobson C.A., Perales M.-A., Kersten M.-J., Oluwole O.O., Ghobadi A., Rapoport A.P., McGuirk J., Pagel J.M. (2021). Axicabtagene Ciloleucel as Second-Line Therapy for Large B-Cell Lymphoma. N. Engl. J. Med..

[10] Bishop M.R., Dickinson M., Purtill D., Barba P., Santoro A., Hamad N., Kato K., Sureda A., Greil R., Thieblemont C. (2022). Second-Line Tisagenlecleucel or Standard Care in Aggressive B-Cell Lymphoma. N. Engl. J. Med..

[11] Wang M., Munoz J., Goy A., Locke F.L., Jacobson C.A., Hill B.T., Timmerman J.M., Holmes H., Jaglowski S., Flinn I.W. (2020). KTE-X19 CAR T-Cell Therapy in Relapsed or Refractory Mantle-Cell Lymphoma. N. Engl. J. Med..

[12] Kontermann R.E., Brinkmann U. (2015). Bispecific antibodies. Drug Discov. Today.

[13] Duell J., Lammers P.E., Djuretic I., Chunyk A.G., Alekar S., Jacobs I., Gill S. (2019). Bispecific Antibodies in the Treatment of Hematologic Malignancies. Clin. Pharmacol. Ther..

[14] Fangazio M., Ladewig E., Gomez K., Garcia-Ibanez L., Kumar R., Teruya-Feldstein J., Rossi D., Filip I., Pan-Hammarström Q., Inghirami G. (2021). Genetic mechanisms of HLA-I loss and immune escape in diffuse large B cell lymphoma. Proc. Natl. Acad. Sci. USA.

[15] Singh A., Dees S., Grewal I.S. (2021). Overcoming the challenges associated with CD3+ T-cell redirection in cancer. Br. J. Cancer.

[16] Falchi L., Vardhana S.A., Salles G.A. (2023). Bispecific antibodies for the treatment of B-cell lymphoma: Promises, unknowns, and opportunities. Blood.

[17] Tavarozzi R., Manzato E. (2022). The Role of Bispecific Antibodies in Non-Hodgkin’s Lymphoma: From Structure to Prospective Clinical Use. Antibodies.

[18] Bates A., Power C.A. (2019). David vs. Goliath: The Structure, Function, and Clinical Prospects of Antibody Fragments. Antibodies.

[19] Papageorgiou S.G., Thomopoulos T.P., Liaskas A., Vassilakopoulos T.P. (2022). Monoclonal Antibodies in the Treatment of Diffuse Large B-Cell Lymphoma: Moving beyond Rituximab. Cancers.

[20] Li H., Er Saw P., Song E. (2020). Challenges and strategies for next-generation bispecific antibody-based antitumor therapeutics. Cell Mol. Immunol..

[21] Salvaris R., Ong J., Gregory G.P. (2021). Bispecific Antibodies: A Review of Development, Clinical Efficacy and Toxicity in B-Cell Lymphomas. J. Pers. Med..

[22] Kuchnio A., Yang D., Vloemans N., Lowenstein C., Cornelissen I., Amorim R., Han C., Sukumaran S., Janssen L., Suls T. (2022). Characterization of JNJ-80948543, a Novel CD79bxCD20xCD3 Trispecific T-Cell Redirecting Antibody for the Treatment of B-Cell Non-Hodgkin Lymphoma. Blood.

[23] Lu H., Oka A., Coulson M., Polli J.R., Aardalen K., Ramones M., Walker D.B., Carrion A., Alexander D., Klopfenstein M. (2022). PIT565, a First-in-Class Anti-CD19, Anti-CD3, Anti-CD2 Trispecific Antibody for the Treatment of B Cell Malignancies. Blood.

[24] Kantarjian H., Stein A., Gökbuget N., Fielding A.K., Schuh A.C., Ribera J.M., Wei A., Dombret H., Foà R., Bassan R. (2017). Blinatumomab versus Chemotherapy for Advanced Acute Lymphoblastic Leukemia. N. Engl. J. Med..

[25] Gokbuget N., Dombret H., Bonifacio M., Reichle A., Graux C., Faul C., Diedrich H., Topp M.S., Bruggemann M., Horst H.A. (2018). Blinatumomab for minimal residual disease in adults with B-cell precursor acute lymphoblastic leukemia. Blood.

[26] Dufner V., Sayehli C.M., Chatterjee M., Hummel H.D., Gelbrich G., Bargou R.C., Goebeler M.E. (2019). Long-term outcome of patients with relapsed/refractory B-cell non-Hodgkin lymphoma treated with blinatumomab. Blood Adv..

[27] Coyle L., Morley N.J., Rambaldi A., Mason K.D., Verhoef G., Furness C.L., Zhang A., Jung A.S., Cohan D., Franklin J.L. (2020). Open-Label, phase 2 study of blinatumomab as second salvage therapy in adults with relapsed/refractory aggressive B-cell non-Hodgkin lymphoma. Leuk Lymphoma.

[28] Viardot A., Goebeler M.E., Hess G., Neumann S., Pfreundschuh M., Adrian N., Zettl F., Libicher M., Sayehli C., Stieglmaier J. (2016). Phase 2 study of the bispecific T-cell engager (BiTE) antibody blinatumomab in relapsed/refractory diffuse large B-cell lymphoma. Blood.

[29] Poh C., Frankel P., Ruel C., Abedi M., Schwab E., Costello C.L., Zain J., Budde L.E., William B.M., Foss F.M. (2019). Blinatumomab/Lenalidomide in Relapsed/Refractory Non-Hodgkin’s Lymphoma: A Phase I California Cancer Consortium Study of Safety, Efficacy and Immune Correlative Analysis. Blood.

[30] Ghobadi A., Rettig M., Cashen A., Gehrs L., Christ S., Mehta-Shah N., Westervelt P., Kahl B., Bartlett N., Dipersio J. (2020). Blinatumomab Consolidation Post Autologous Hematopoietic Stem Cell Transplantation in Patients with Diffuse Large B Cell Lymphoma. Blood.

[31] Budde L.E., Assouline S., Sehn L.H., Schuster S.J., Yoon S.S., Yoon D.H., Matasar M.J., Bosch F., Kim W.S., Nastoupil L.J. (2022). Single-Agent Mosunetuzumab Shows Durable Complete Responses in Patients with Relapsed or Refractory B-Cell Lymphomas: Phase I Dose-Escalation Study. J. Clin. Oncol..

[32] Hutchings M., Mous R., Clausen M.R., Johnson P., Linton K.M., Chamuleau M.E.D., Lewis D.J., Sureda Balari A., Cunningham D., Oliveri R.S. (2021). Dose escalation of subcutaneous epcoritamab in patients with relapsed or refractory B-cell non-Hodgkin lymphoma: An open-label, phase 1/2 study. Lancet.

[33] Thieblemont C., Phillips T., Ghesquieres H., Cheah C.Y., Clausen M.R., Cunningham D., Do Y.R., Feldman T., Gasiorowski R., Jurczak W. (2023). Epcoritamab, a Novel, Subcutaneous CD3xCD20 Bispecific T-Cell-Engaging Antibody, in Relapsed or Refractory Large B-Cell Lymphoma: Dose Expansion in a Phase I/II Trial. J. Clin. Oncol..

[34] Thieblemont C., Karimi Y., Jurczak W., Cheah C.Y., Clausen M.R., Cunningham D., Do Y.R., Lewis D.J., Gasiorowski R., Kim T.M. (2023). Subcutaneous epcoritamab induces deep, durable complete remissions in relapsed/refractory large B-cell lymphoma: Longer follow-up from the pivotal epcore NHL-1 trial. Hematol. Oncol..

[35] Bacac M., Colombetti S., Herter S., Sam J., Perro M., Chen S., Bianchi R., Richard M., Schoenle A., Nicolini V. (2018). CD20-TCB with Obinutuzumab Pretreatment as Next-Generation Treatment of Hematologic Malignancies. Clin. Cancer Res..

[36] Dickinson M.J., Carlo-Stella C., Morschhauser F., Bachy E., Corradini P., Iacoboni G., Khan C., Wrobel T., Offner F., Trneny M. (2022). Glofitamab for Relapsed or Refractory Diffuse Large B-Cell Lymphoma. N. Engl. J. Med..

[37] Hutchings M., Avigdor A., Sureda A., Terol M.J., Bosch F., Corradini P., Larsen T.S., Domínguez A.R., Skarbnik A., Jørgensen J. (2023). Glofitamab plus polatuzumab vedotin demonstrates durable responses and a manageable safety profile in patients with relapsed/refractory diffuse large B-cell lymphoma. Hematol. Oncol..

[38] Smith E.J., Olson K., Haber L.J., Varghese B., Duramad P., Tustian A.D., Oyejide A., Kirshner J.R., Canova L., Menon J. (2015). A novel, native-format bispecific antibody triggering T-cell killing of B-cells is robustly active in mouse tumor models and cynomolgus monkeys. Sci. Rep..

[39] Bannerji R., Arnason J.E., Advani R.H., Brown J.R., Allan J.N., Ansell S.M., Barnes J.A., O’Brien S.M., Chavez J.C., Duell J. (2022). Odronextamab, a human CD20xCD3 bispecific antibody in patients with CD20-positive B-cell malignancies (ELM-1): Results from the relapsed or refractory non-Hodgkin lymphoma cohort in a single-arm, multicentre, phase 1 trial. Lancet Haematol..

[40] Poon M., Walewski J., Kim T.M., Cho S., Jarque I., Iskierka-Jażdżewska E., Prince H.M., Oh S.Y., Lim F., Carpio C. (2023). Odronextamab in patients with relapsed/refractory diffuse large B-cell lymphoma (DLBCL): Results from a prespecified analysis of the pivotal phase II study ELM-2. Hematol. Oncol..

[41] Patel K., Riedell P.A., Tilly H., Ahmed S., Michot J.-M., Ghesquieres H., Schiano de Collela J.M., Chanan-Khan A., Bouabdallah K., Tessoulin B. (2022). A Phase 1 Study of Plamotamab, an Anti-CD20 x Anti-CD3 Bispecific Antibody, in Patients with Relapsed/Refractory Non-Hodgkin’s Lymphoma: Recommended Dose Safety/Efficacy Update and Escalation Exposure-Response Analysis. Blood.

[42] Patel K., Koh Y., Ayyappan S., Karimi Y., Lossos I.S., Merchant A., Lee P., Jin J., Clynes R., Kanodia J. (2022). Phase 2 Randomized, Open-Label, Multicenter Study to Evaluate the Efficacy and Safety of Plamotamab Combined with Tafasitamab (Tafa) + Lenalidomide (Len) Vs Tafa+Len in Relapsed or Refractory DLBCL. Blood.

[43] Tilly H., Morschhauser F., Sehn L.H., Friedberg J.W., Trneny M., Sharman J.P., Herbaux C., Burke J.M., Matasar M., Rai S. (2022). Polatuzumab Vedotin in Previously Untreated Diffuse Large B-Cell Lymphoma. N. Engl. J. Med..

[44] Kamdar M., Solomon S.R., Arnason J., Johnston P.B., Glass B., Bachanova V., Ibrahimi S., Mielke S., Mutsaers P., Hernandez-Ilizaliturri F. (2022). Lisocabtagene maraleucel versus standard of care with salvage chemotherapy followed by autologous stem cell transplantation as second-line treatment in patients with relapsed or refractory large B-cell lymphoma (TRANSFORM): Results from an interim analysis of an open-label, randomised, phase 3 trial. Lancet.

[45] Lee D.W., Gardner R., Porter D.L., Louis C.U., Ahmed N., Jensen M., Grupp S.A., Mackall C.L. (2014). Current concepts in the diagnosis and management of cytokine release syndrome. Blood.

[46] Lee D.W., Santomasso B.D., Locke F.L., Ghobadi A., Turtle C.J., Brudno J.N., Maus M.V., Park J.H., Mead E., Pavletic S. (2019). ASTCT Consensus Grading for Cytokine Release Syndrome and Neurologic Toxicity Associated with Immune Effector Cells. Biol. Blood Marrow Transpl..

[47] Shimabukuro-Vornhagen A., Godel P., Subklewe M., Stemmler H.J., Schlosser H.A., Schlaak M., Kochanek M., Boll B., von Bergwelt-Baildon M.S. (2018). Cytokine release syndrome. J. Immunother. Cancer.

[48] Winkler U., Jensen M., Manzke O., Schulz H., Diehl V., Engert A. (1999). Cytokine-release syndrome in patients with B-cell chronic lymphocytic leukemia and high lymphocyte counts after treatment with an anti-CD20 monoclonal antibody (rituximab, IDEC-C2B8). Blood.

[49] Brudno J.N., Kochenderfer J.N. (2016). Toxicities of chimeric antigen receptor T cells: Recognition and management. Blood.

[50] Maude S.L., Teachey D.T., Porter D.L., Grupp S.A. (2015). CD19-targeted chimeric antigen receptor T-cell therapy for acute lymphoblastic leukemia. Blood.

[51] Hutchings M., Morschhauser F., Iacoboni G., Carlo-Stella C., Offner F.C., Sureda A., Salles G., Martinez-Lopez J., Crump M., Thomas D.N. (2021). Glofitamab, a Novel, Bivalent CD20-Targeting T-Cell-Engaging Bispecific Antibody, Induces Durable Complete Remissions in Relapsed or Refractory B-Cell Lymphoma: A Phase I Trial. J. Clin. Oncol..

[52] Gust J., Hay K.A., Hanafi L.A., Li D., Myerson D., Gonzalez-Cuyar L.F., Yeung C., Liles W.C., Wurfel M., Lopez J.A. (2017). Endothelial Activation and Blood-Brain Barrier Disruption in Neurotoxicity after Adoptive Immunotherapy with CD19 CAR-T Cells. Cancer Discov..

[53] Santomasso B.D., Park J.H., Salloum D., Riviere I., Flynn J., Mead E., Halton E., Wang X., Senechal B., Purdon T. (2018). Clinical and Biological Correlates of Neurotoxicity Associated with CAR T-cell Therapy in Patients with B-cell Acute Lymphoblastic Leukemia. Cancer Discov..

[54] Kennedy A.D., Beum P.V., Solga M.D., DiLillo D.J., Lindorfer M.A., Hess C.E., Densmore J.J., Williams M.E., Taylor R.P. (2004). Rituximab infusion promotes rapid complement depletion and acute CD20 loss in chronic lymphocytic leukemia. J. Immunol..

[55] Foran J.M., Norton A.J., Micallef I.N., Taussig D.C., Amess J.A., Rohatiner A.Z., Lister T.A. (2001). Loss of CD20 expression following treatment with rituximab (chimaeric monoclonal anti-CD20): A retrospective cohort analysis. Br. J. Haematol..

[56] Brouwer-Visser J., Fiaschi N., Deering R., Dhanik A., Cygan K., Zhang W., Jeong S., Pourpe S., Boucher L., Hamon S. (2020). Baseline Biomarkers of T-Cell Function Correlate with Clinical Responses to Odronextamab (REGN1979), and Loss of CD20 Target Antigen Expression Identified As a Mechanism of Treatment Resistance. Blood.

[57] Broske A.E., Korfi K., Belousov A., Wilson S., Ooi C.H., Bolen C.R., Canamero M., Alcaide E.G., James I., Piccione E.C. (2022). Pharmacodynamics and molecular correlates of response to glofitamab in relapsed/refractory non-Hodgkin lymphoma. Blood Adv..

[58] Pascual M., Mena-Varas M., Robles E.F., Garcia-Barchino M.J., Panizo C., Hervas-Stubbs S., Alignani D., Sagardoy A., Martinez-Ferrandis J.I., Bunting K.L. (2019). PD-1/PD-L1 immune checkpoint and p53 loss facilitate tumor progression in activated B-cell diffuse large B-cell lymphomas. Blood.

[59] Shouval R., Alarcon Tomas A., Fein J.A., Flynn J.R., Markovits E., Mayer S., Olaide Afuye A., Alperovich A., Anagnostou T., Besser M.J. (2022). Impact of TP53 Genomic Alterations in Large B-Cell Lymphoma Treated with CD19-Chimeric Antigen Receptor T-Cell Therapy. J. Clin. Oncol..

[60] God J.M., Cameron C., Figueroa J., Amria S., Hossain A., Kempkes B., Bornkamm G.W., Stuart R.K., Blum J.S., Haque A. (2015). Elevation of c-MYC disrupts HLA class II-mediated immune recognition of human B cell tumors. J. Immunol..

[61] Budde E., Gopal A.K., Kim W.S., Flinn I.W., Cheah C.Y.Y., Nastoupil L., Matasar M.J., Diefenbach C.S., Gregory G.P., Qazi I. (2021). A Phase 1 Dose Escalation Study of Igm-2323, a Novel Anti-CD20 x Anti-CD3 IgM T Cell Engager (TCE) in Patients with Advanced B-Cell Malignancies. Blood.

